# Surgical treatment of cervical spondylosis in patients 80 years of age and older—A retrospective observational study

**DOI:** 10.1371/journal.pone.0217725

**Published:** 2019-06-26

**Authors:** Kiyoshi Ito, Takuya Nakamura, Tetsuyoshi Horiuchi, Kazuhiro Hongo

**Affiliations:** Department of Neurosurgery, Shinshu University School of Medicine, Matsumoto, Japan; Universita degli Studi di Palermo, ITALY

## Abstract

The older adult population in developed countries is rapidly increasing, as is the number of older adults with cervical spondylosis. Previous studies on the surgical outcomes of older adults with cervical spondylosis have reported contradictory results. This study aimed to compare the surgical outcomes in adults with cervical spondylosis who were <80 and ≥80 years old. We retrospectively investigated data from adults who underwent surgical treatment for cervical spondylosis between 2006 and 2016. The clinical outcomes and postoperative complications of patients who were <80 years old were compared to those of patients who were ≥80 years old. Of the 108 patients included in the study, 14 (13.0%) were ≥80 years old. The preoperative neurosurgical cervical spine score was significantly different between patients who were <80 (9.1 ± 2.4) and ≥80 (6.1 ± 2.1) years old (p < .001). The recovery rate was 58.2 ± 30.0% and 41.3 ± 24.7% in patients who were <80 and ≥80 years old, respectively (p = .05). However, the number of recovery points scored was 2.8 ± 2.0 and 3.4 ± 2.3 in patients who were <80 and ≥80 years old, respectively, which was not significantly different. Although 12 patients had medical comorbidities, they had no surgical complications. This study clarifies the benefits of surgical treatment for older adults with cervical spondylosis. Generally, older adults have lower recovery rates and are unlikely to experience full recovery; however, surgery for cervical spondylosis appears to improve patients’ quality of life.

## Introduction

The older adult population is increasing in most developed countries. According to a survey conducted by the Japanese Ministry of Health, Labor, and Welfare, the population of individuals 80 years of age or older in Japan was more than 10,020,000 in 2017, which accounted for 7.9% of the entire population.

Cervical spondylosis is an age-related neurological disorder caused by narrowing of the cervical spinal canal owing to degeneration of the intervertebral discs and adjacent vertebral structures. The number of older adults with cervical spondylosis is rapidly increasing in developed countries. Although neurospinal surgeons occasionally need to manage such patients, treatment strategies for older adults remain controversial due to the age-related and systemic complications of surgery [1,2].

In the present study, we evaluated the clinical features, surgical outcomes, and age-related surgical complications of patients who underwent surgery for cervical spondylosis at our hospital between 2006 and 2016 and compared the findings between patients who were <80 and ≥80 years of age. We think that our results will provide insight into the optimal surgical strategies for older adults with cervical spondylosis.

## Materials and methods

Written informed consent was obtained from all individuals included in the study. The study was conducted according to the principles expressed in the Declaration of Helsinki and the protocol was approved by our Shinshu University Institutional Review Board (No. 4034).

### Patient population

A total of 108 patients with cervical spondylosis were surgically treated in our hospital between 2006 and 2016. These patients comprised 79 men and 29 women, with a mean age of 63.8 years (range: 25–88 years) at the time of surgery. The observation period ranged from 3 to 108 months (mean: 26.1 months) [S1 Dataset].

### Selection of surgical procedures

To be eligible for the study, the patients were required to be able to tolerate general anesthesia. An anterior surgical approach was predominantly selected because it allowed for removal of the lesion directly from the anterior side. In our limited experience, this approach is adequate for surgeries involving one or two affected disc levels. For surgeries involving more than three levels, including ossification of the posterior longitudinal ligament and lordosis shape of the spine, we performed an expansive laminoplasty with ceramic spacers for decompression. Preoperative neuroimaging was used to determine the appropriate surgical approach.

### Assessment of postoperative surgical outcome, preoperative medical comorbidities, and related complications

**Fig 1:**
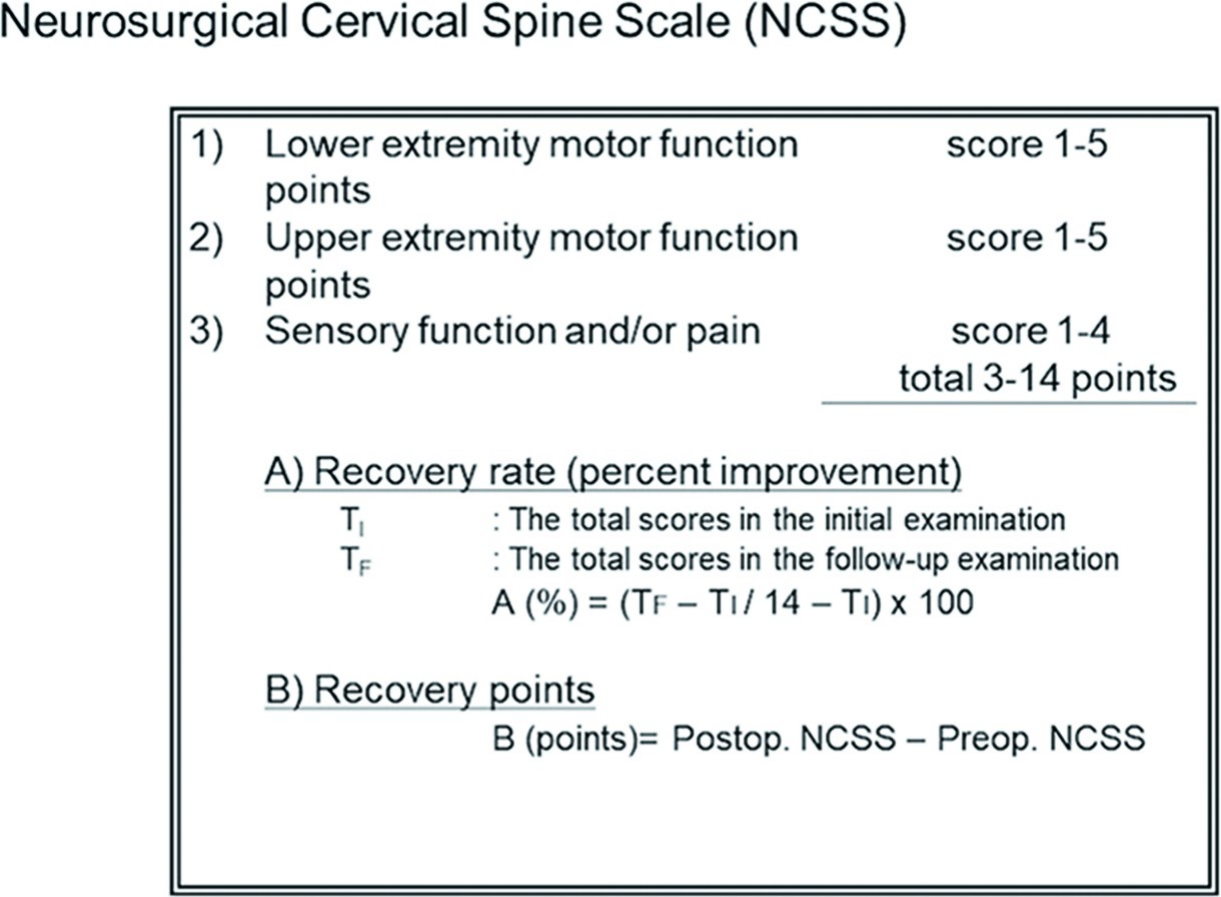
The neurosurgical cervical spine scale (NCSS) grading and scoring system for neurological function in degenerative cervical spine disease. On this scale, healthy individuals receive a score of 14 points. This scale is divided into three categories evaluating motor and sensory function. The higher the NCSS score, the better the functional status of the patient at the time of the assessment. The recovery rate (%) indicates the percent improvement of the patient. In order to clearly understand the patients’ characteristics, “recovery points (points)” were also used in this study. Postop., postoperative; Preop., preoperative.

We evaluated the patients’ perioperative clinical symptoms according to the neurosurgical cervical spine scale (NCSS) proposed by the Neurospinal Society of Japan [3]. On the NCSS, healthy individuals score 14 points (Fig 1). The NCSS is divided into three categories (motor function of the lower extremities, motor function of the upper extremities, and sensory function), and is assessed according to a patient’s condition.

The NCSS score and recovery rate (%) were used to calculate the mean difference in preoperative and postoperative status. The higher the NCSS score, the better the functional status of the patient at the time of the assessment. The recovery rate (%), described by Kadoya, et al. [3], indicating the degree of recovery of normal function postoperatively, was calculated using the following formula: (postoperative NCSS score–preoperative NCSS score) / (14 –preoperative NCSS score) × 100.

In order to clearly understand the patients’ characteristics, we defined and calculated a measure called “recovery points.” Recovery points were calculated using the following formula: (postoperative NCSS score–preoperative NCSS score).

Additionally, patients’ preoperative medical comorbidities and related complications were evaluated.

### Statistical analysis

Data were analyzed using the Wilcoxon signed-rank test for the assessment of perioperative surgical outcomes and the chi-squared test for the assessment of surgical selection. All statistical calculations were performed using Jump Statistics, version 12.2 (SAS Institute Inc., Cary, NC). Differences were considered statistically significant at p < .05.

## Results

### Assessment of patient distribution by age, sex, and percentage of patients ≥80 years of age

**Fig 2:**
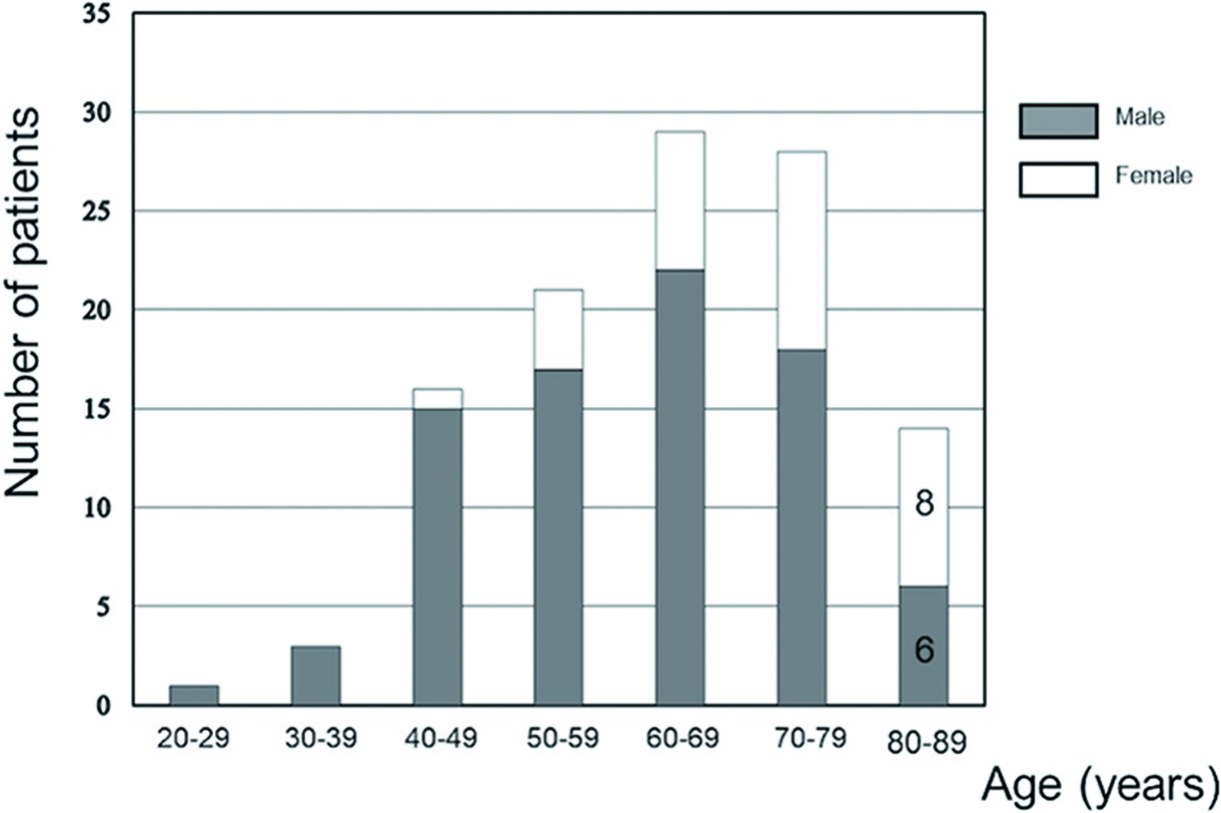
Bar graph showing the distribution of patients who underwent surgery for cervical spondylosis by age and sex. There were more female than male patients over the age of 80 years in the population.

**Fig 3:**
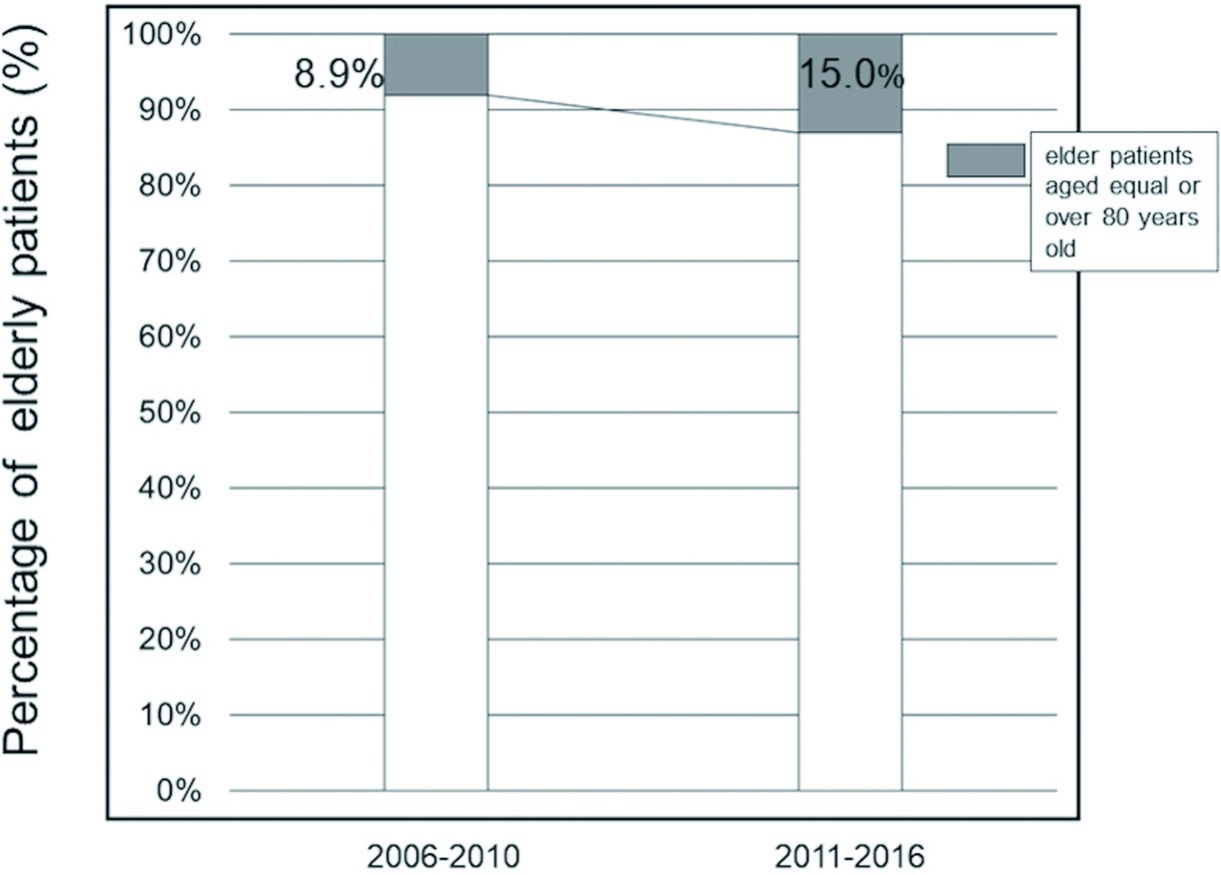
Bar graph showing the percentage of patients in the study aged ≥80 years.

Of the 108 patients treated for cervical spondylosis during the 11-year period spanning from 2006 to 2016, 14 (13.0%) were aged ≥80 years. Fig 2 shows the age and sex distribution of the patients. The number of patients ≥80 years who underwent surgical treatment gradually increased from 2006 to 2016 (Fig 3).

### Surgical selection

**Fig 4:**
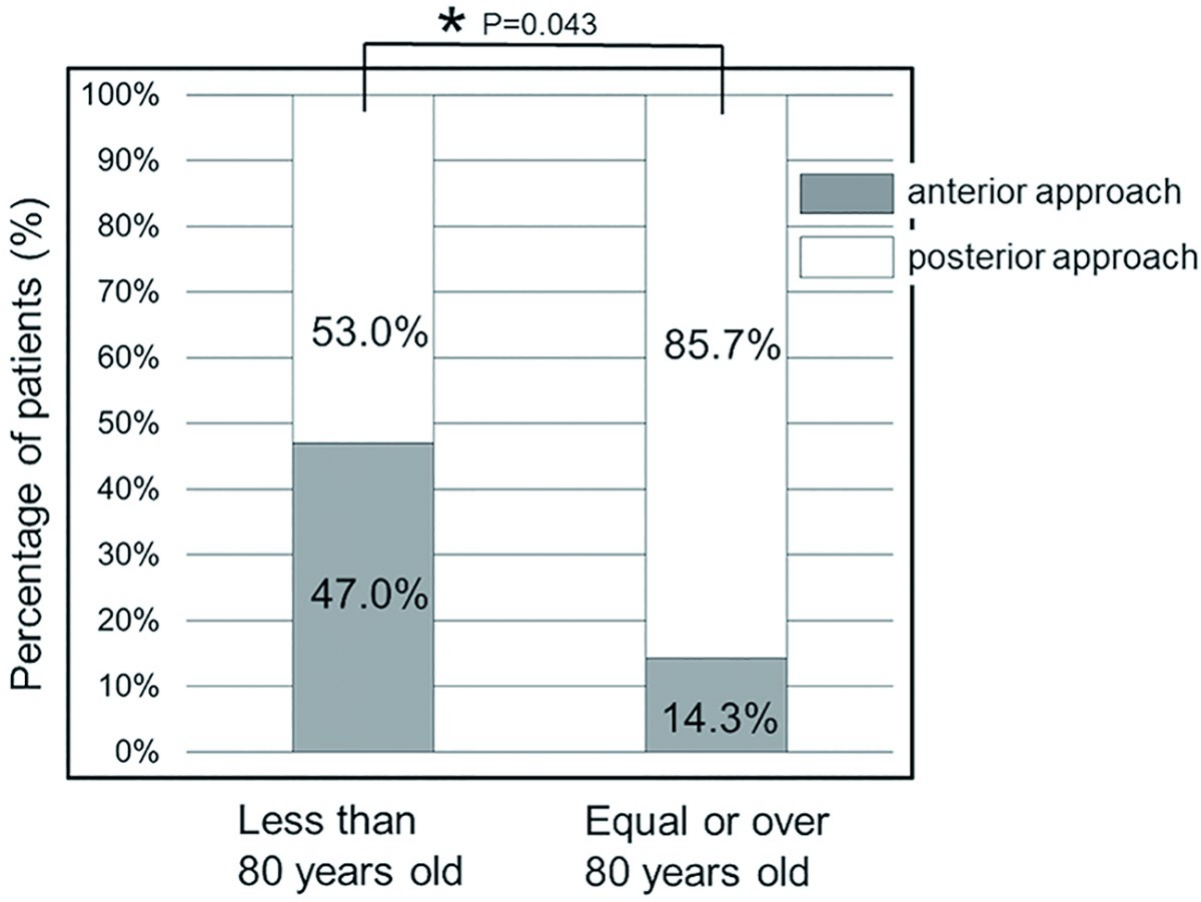
Bar graph showing the proportion of patients in the study treated using anterior and posterior surgical approaches.

Among patients <80 years old, 53.0% were treated with expansive laminoplasty and 47.0% with an anterior approach (Fig 4). Alternately, laminoplasty was performed in 85.7% of patients ≥80 years of age. The two patient groups were significantly different with regard to the proportion of surgery types performed (p = .0043).

### Assessment of perioperative surgical outcomes

**Fig 5:**
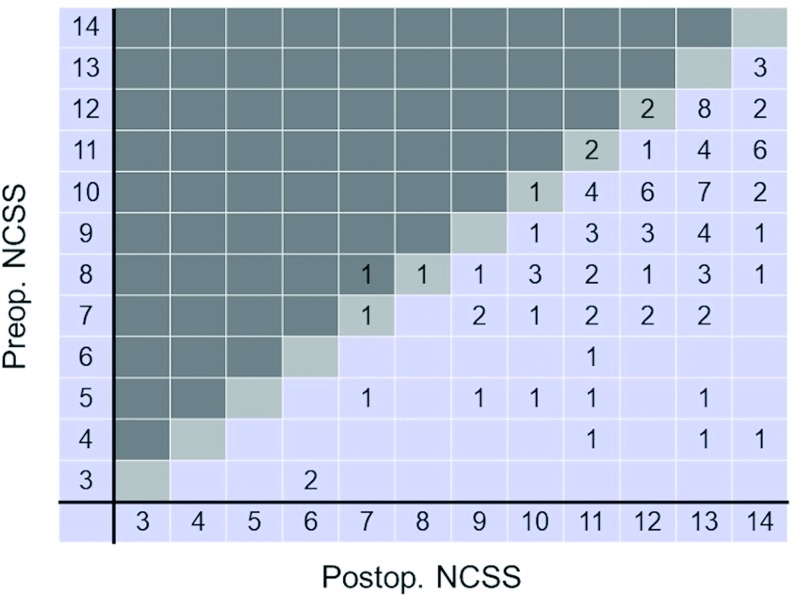
Improvement in the neurosurgical cervical spine scale (NCSS) scores of patients <80 years of age. Postoperatively, the majority of patients scored >11 points. Fourteen patients achieved the full score (14 points) postoperatively, indicating complete improvement in their preoperative symptoms (i.e., 100% recovery rate). Postop., postoperative; Preop., preoperative.

**Fig 6:**
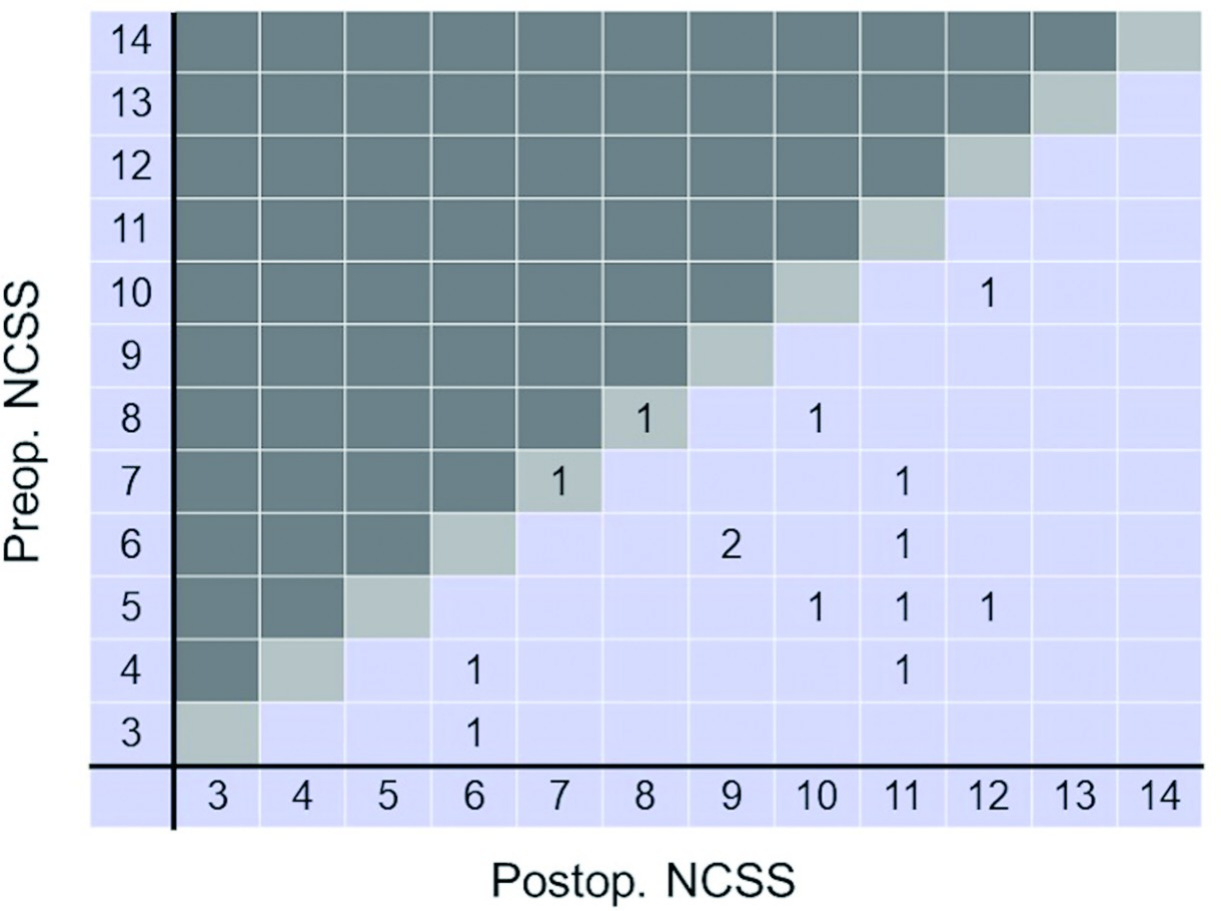
The improvement in the neurosurgical cervical spine scale (NCSS) scores of patients aged ≥80 years. Postoperatively, the majority of patients scored up to 9 points. None of the patients achieved a full score (14 points) postoperatively. Most patients, however, did recover to some extent following surgery. Postop., postoperative; Preop., preoperative.

Of the 108 patients with cervical spondylosis, all had their functional status evaluated before and after surgery using the NCSS. Sixteen of the patients <80 years old recovered completely after surgery (Fig 5). However, none of the patients ≥80 years of age recovered completely after surgery (Fig 6).

The preoperative NCSS score was significantly different between patients <80 and ≥80 years old (Table 1, p < .001). Patients ≥80 years old had a significantly lower recovery rate than did patients <80 years old (p = .05). The number of recovery points scored was not significantly different between patients <80 and ≥80 years old (Table 1, p = .27).

**Table 1:** Comparisons of the perioperative clinical evaluations between the two patient groups.

	<80 years of age	≥80 years of age	p value
**Preoperative NCSS**	9.1 ± 2.4	6.1 ± 2.1	< .001
**Postoperative NCSS**	11.9 ± 2.0	9.5 ± 2.1	< .001
**Recovery rate (%)**	58.2 ± 29.9	41.3 ± 24.7	.05
**Recovery points (points)**	2.8 ± 2.0	3.4 ± 2.3	.27

NCSS, neurosurgical cervical spine scale.

### Assessment of preoperative medical comorbidities and surgery-related complications

Preoperatively, patients ≥80 years old had medical comorbidities that had to be managed carefully during the perioperative period. Specifically, of the 14 patients ≥80 years old, 12 had comorbid medical conditions. The comorbidities included five cases of hypertension, five cases of diabetes mellitus, two cases of dyslipidemia, and one case each of arrhythmia, prostate cancer without metastasis, and atherosclerosis obliterans. Fortunately, these comorbidities did not affect postoperative complications because of the intensive postoperative treatments. Additionally, there were no procedure-related or comorbid medical condition-related complications.

## Discussion

According to these results, although patients ≥80 years of age are unlikely to experience complete recovery following surgery, it may be possible to achieve an improvement in their quality of life. The results of this study should be considered when managing patients ≥80 years old with cervical lesions.

Previous studies have reported on the surgical outcomes of older adults with cervical spondylosis. Several reports have indicated poorer surgical outcomes in older than in younger adults [4–7]. Other studies did not find any significant differences in surgical outcomes between older and younger adults [1,8]. In this study, the preoperative NCSS score of patients ≥80 years old was significantly lower than that of patients <80 years old. Previous reports have shown that older adults tend to experience longer symptom durations prior to surgery for cervical spondylosis [8]. Moreover, it is important to note that lower NCSS scores are not solely reflective of myelopathy, but also of other comorbidities, such as hip and knee osteoarthritis, cerebrovascular disease, and diabetic neuropathy [2,9]. Further, when a surgical approach is selected, it should be considered that older adults might have multiple and severe cord compressions; therefore, the preoperative NCSS score might be lower in older patients than in younger patients. Here, the proportion of patients for whom a posterior surgical approach for multiple segment stenosis vs. an anterior approach was used was significantly higher in patients ≥80 years old. These findings suggest that the preoperative status of patients ≥80 years old is much more severe than that of younger patients. That being said, the NCSS score has several pitfalls, as does the recovery rate of the NCSS score. The most crucial scientific limitation is the fact that the actual surgical outcomes of patients with the same recovery rate may differ as a result of differing preoperative NCSS scores. Since the recovery rate of the NCSS score indicates the percent improvement of the patient, lower preoperative NCSS scores result in lower recovery rate scores.

In lieu of the recovery rate of the NCSS score, we developed and used a measure of “recovery points,” which was calculated as the difference between the preoperative and postoperative NCSS scores. The recovery rate of the NCSS score was lower in patients ≥80 years old than in patients <80 years old, but the recovery points were not significantly different between the two groups. Moreover, there were no postoperative complications influenced by medical comorbidities in patients ≥80 years old. Thus, although older adults are less likely to experience complete recovery following surgery, our results indicate that surgery may improve their quality of life.

Our results suggest that, even in older adults, surgery is an acceptable means by which to improve the symptoms caused by cervical spondylosis. It is well known that early surgical intervention is associated with better outcomes in older adults [10]. Thus, it is necessary for older adults to undergo surgery for cervical spondylosis. Moreover, there is a possibility that early surgical intervention can improve the postoperative outcomes of older adults, even in those with low preoperative NCSS scores.

Our study had some limitations. The presence of medical comorbidities may render a patient unable to tolerate general anesthesia. Among the patients ≥80 years old who were considered for this study, some were deemed poor candidates for surgery due to severe systemic issues. Therefore, patient selection may have been a limitation of this study.

Although patients ≥80 years of age are unlikely to experience complete recovery following surgery, it may be possible to achieve an improvement in their quality of life. Moreover, it is important to manage older adults carefully in order to prevent medical comorbidities and complications perioperatively. Altogether, the results of this study should be considered when managing patients ≥80 years old with cervical lesions.

## Supporting information

S1 Dataset This date-set is on age over 80 years old / under 80 years old patients, pathology (CS:cervical spondylosis), procedure, NCSS score before the operation, its total, NCSS score after the operation, its total, improved score and improved rate. (XLSX)
